# T4 RNA Ligase 2 truncated active site mutants: improved tools for RNA analysis

**DOI:** 10.1186/1472-6750-11-72

**Published:** 2011-07-01

**Authors:** Sebastien Viollet, Ryan T Fuchs, Daniela B Munafo, Fanglei Zhuang, Gregory B Robb

**Affiliations:** 1 New England Biolabs Inc. 240 County Road, Ipswich, MA 02143, USA

## Abstract

**Background:**

T4 RNA ligases 1 and 2 are useful tools for RNA analysis. Their use upstream of RNA analyses such as high-throughput RNA sequencing and microarrays has recently increased their importance. The truncated form of T4 RNA ligase 2, comprising amino acids 1-249 (T4 Rnl2tr), is an attractive tool for attachment of adapters or labels to RNA 3'-ends. Compared to T4 RNA ligase 1, T4 Rnl2tr has a decreased ability to ligate 5'-PO_4 _ends in single-stranded RNA ligations, and compared to the full-length T4 Rnl2, the T4 Rnl2tr has an increased activity for joining 5'-adenylated adapters to RNA 3'-ends. The combination of these properties allows adapter attachment to RNA 3'-ends with reduced circularization and concatemerization of substrate RNA.

**Results:**

With the aim of further reducing unwanted side ligation products, we substituted active site residues, known to be important for adenylyltransferase steps of the ligation reaction, in the context of T4 Rnl2tr. We characterized the variant ligases for the formation of unwanted ligation side products and for activity in the strand-joining reaction.

**Conclusions:**

Our data demonstrate that lysine 227 is a key residue facilitating adenylyl transfer from adenylated ligation donor substrates to the ligase. This reversal of the second step of the ligation reaction correlates with the formation of unwanted ligation products. Thus, T4 Rn2tr mutants containing the K227Q mutation are useful for reducing undesired ligation products. We furthermore report optimal conditions for the use of these improved T4 Rnl2tr variants.

## Background

RNA ligases have become useful tools to label, circularize, or to perform intermolecular ligation of RNA [1,2]. Bacteriophage T4 encodes 2 proteins with ligase activity: T4 RNA ligase 1 (T4 Rnl1), and the more recently discovered T4 RNA ligase 2 (T4 Rnl2) [3].

**Figure 1:**
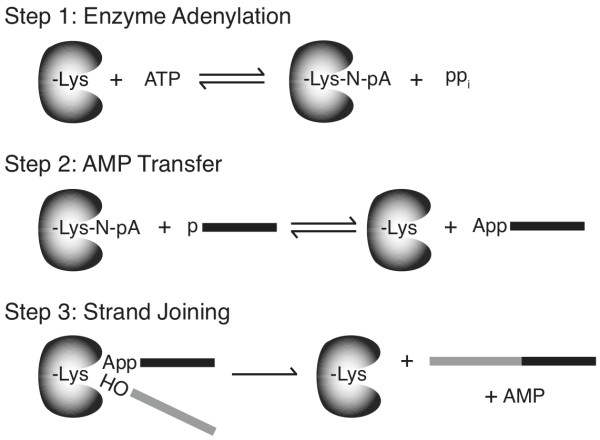
**Schematic representation of a nucleic acid ligation reaction**. In step 1, the enzyme reacts with ATP and becomes adenylated on an active site lysine residue yielding adenylated enzyme and pyrophosphate. In step 2, the AMP is transferred from the active site lysine to a 5'-phosphorylated nucleic acid donor (black). In step 3, the enzyme promotes phosphodiester bond formation between the 5'-adenylated nucleic acid donor, and a polynucleotide acceptor molecule that is 3'-hydroxylated (grey). The reaction yields a ligated polynucleotide and AMP.

The T4 RNA ligases are able to join adjacent 3'-OH and 5'-PO_4 _polynucleotides as a result of a three-step reaction (Figure 1). In the first step, the enzyme becomes adenylated on an active site lysine generating an AMP-ligase intermediate and inorganic pyrophosphate. In the second step, the AMP is transferred from the enzyme to 5'-PO_4 _RNA to produce a 5'-adenylated polynucleotide. The final step is the formation of a phosphodiester bond between the 3'-OH ligation acceptor and 5'-adenylated polynucleotide ligation donor. The reaction is promoted by the attack on the 5'-phosphorus by the 3'-OH and results in AMP release [4].

During a ligation reaction, T4 RNA ligases have the potential to produce undesired products such as concatemers or circles. These products arise due to the presence of ATP and polynucleotides with 5'-PO_4_, and 3'-OH. After steps 1 and 2 of the ligation reaction, the resulting 5'-adenylated polynucleotide has the ability to ligate to another 3'-OH polynucleotide or to its own 3'-OH end. These ligation side products may be troublesome for analysis and quantification.

Extensive structural and functional studies of T4 Rnl2 have established roles for domains and key residues necessary for ligation activity of the enzyme. For instance, the N-terminal domain (1-243) is essential for the ligase adenylation (step1) and for the phosphodiester bond formation (step 3) while the C-terminal (244-329) domain is dispensable for these steps, but is involved in the transfer of AMP from the enzyme-AMP complex to 5'-PO_4 _donor [4-6]. Interestingly T4 Rnl2 (1-249) (T4 Rnl2tr) is ten times more active than the full length T4 Rnl2 in 5'-adenylated RNA ligation [5]. These features make T4 Rnl2tr an attractive tool for attaching adapters or labels to RNA 3'-ends due to the inability of the enzyme to adenylate 5'-PO_4 _donor molecules which should reduce the formation of unwanted concatemers in ligation reactions.

Three Rnl2 active site residues (R55, K225 and K227) have properties of interest to us when conservatively substituted in the context of the full length T4 Rnl2. R55K, K227Q, K225R retained the ability to perform ligation step 3 -- phosphodiester bond formation between the 3'-OH RNA and an adenylated RNA [6], yet have decreased ability to form enzyme adenylate complexes (ligation step one).

We wondered whether reducing enzyme-adenylate complex formation in a ligase compromised for donor adenylation would produce fewer unwanted side products even when ligation is performed in the absence of ATP. To answer this question, a series of T4 Rnl2tr mutants was developed incorporating combinations of R55K, K225R and K227Q mutations. The mutants were expressed and purified to characterize their utility for ligation of adenylated oligodeoxynucleotide adapters to small RNAs.

We demonstrate that some enzyme-adenlyation-deficient mutants of the truncated T4 Rnl2 retain the ability to form concatemer side-ligation products. We hypothesize that T4 RNA ligases and truncated ligases form ligation side products because of the reverse reaction of ligation step 2. That is, AMP is transferred from 5'-adenylated adapters to 5'-PO_4 _RNA, thus producing undesired adenylated substrates that form unexpected products by ligation with 3'-OH RNA.

We provide direct evidence for the reversal of ligation reaction step 2 by T4 RNA ligases and correlate that activity with the formation of ligation side-products that include concatemers. Furthermore, we present data that implicate K227 in truncated T4 Rnl2 as key for the reverse ligation reaction step 2. Together these data demonstrate the utility of the K227Q variant of T4 Rnl2tr as a tool for molecular biology, and provide insight into the molecular basis of ligation side product formation.

## Results

### Formation of ligation side products by T4 RNA ligases

**Figure 2:**
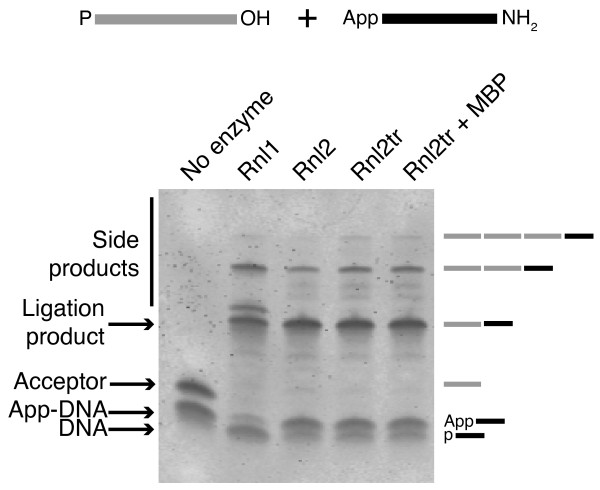
**Production of ligation side products by T4 RNA ligases**. Intermolecular ligation reactions containing 5'-adenylated DNA adapters, 21-mer 5'-PO_4 _RNA acceptors and ligase (1 pmol) were incubated at 16°C overnight with 12.5% PEG 8000. Products of the reactions were resolved on denaturing 15% acrylamide gels and stained with SYBR Gold. The bands corresponding to the input nucleic acids, the DNA adapter/RNA acceptor ligation product (39 bases), and larger side products are indicated. Rnl1 = T4 RNA ligase 1, Rnl2 = T4 RNA ligase 2, Rnl2tr = T4 RNA ligase 2 truncated, Rnl2tr + MBP = T4 RNA ligase 2 truncated attached to an N-terminal maltose binding protein tag. Oligonucleotide substrates are depicted schematically above the gel. Grey lines represent RNA and black lines represent DNA.

Attachment of adapters to RNA 3'-ends is a common first step for RNA quantification and discovery by RT-PCR and high-throughput sequencing. Ligations are commonly performed using an RNA ligase in the absence of ATP to prevent the formation of RNA circles and concatemers [7]. In this case, adapters are 3'- blocked and 5'- adenylated so that they are, in essence, ligation donor reaction intermediates. RNA acceptors are commonly either fragmented from larger RNAs or small RNAs that have 3'-OH and may have 5'-PO_4 _groups. Therefore, promotion of phosphodiester bond formation (step 3) is the only required enzymatic activity for a strand-joining reaction containing an adenylated adapter. We monitored the formation of ligation products using commercially available RNA ligases and defined [5'-PO_4_, 3'-OH] RNA acceptor RNAs and [5'-App, 3'-NH2] DNA donors. In addition to products of the expected size, we detected ligation products that migrated with longer apparent length when treated with Rnl1, Rnl2, or Rnl2tr (Figure 2). This finding was surprising since our ligation reaction conditions should have prevented concatamerization of RNA substrates. The absence of ATP in the reactions should prevent enzyme adenylation in all cases, and the truncation of amino acids 250-346 of T4 Rnl2tr should additionally prevent the transfer of the adenylyl group to any available oligonucleotide 5'-PO_4 _groups.

These observations led us to test the activity of a number of conservative point mutants of T4 Rnl2 when placed in the context of the truncated enzyme. We chose to examine K227Q, K225R, and R55K since previous studies established that these mutations, in the context of the full-length ligase preserved strand-joining activity (phosphodiester bond formation), but were deficient in enzyme adenylation. We reasoned that preventing enzyme adenylation might further reduce the formation of side-ligation products in the context of the truncated ligase. The use of T4 Rnl2tr K227Q for small RNA cloning has been reported [8,9].

### Purification and strand-joining activity of T4 Rnl2tr conservative mutants

**Figure 3:**
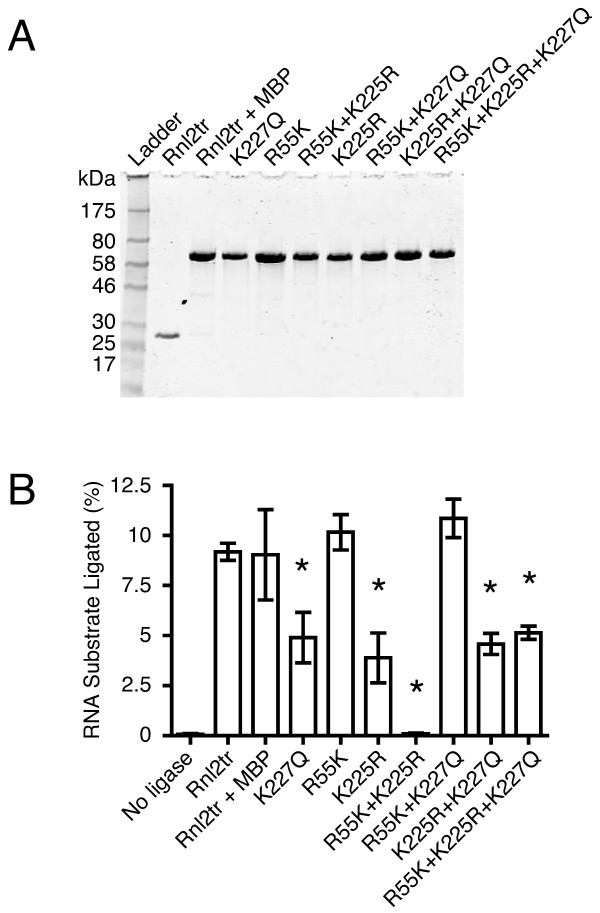
**Purification and activity of T4 RNA Ligase 2 truncated mutants**. **(A) **Aliquots of T4 RNA ligase 2 truncated and mutants were separated on 10-20% Tris-glycine SDS polyacrylamide gels and stained with Coomassie blue. The size (in kDa) of marker polypeptides are indicated on the left. **(B) **Intermolecular strand-joining activity of T4 RNA ligase 2 truncated mutants under multiple turnover conditions. 10 pmol 5'-adenylated 17-mer DNA was incubated for one hour at 25°C with 5 pmol 5'- FAM-labeled 31-mer RNA. 1 pmol of each ligase was added into reaction mixture. The reaction products were resolved on denaturing 15% acrylamide gels, scanned and quantified as described in the methods section. Rnl2tr = T4 RNA ligase 2 truncated, Rnl2tr + MBP = T4 RNA ligase 2 truncated attached to an N-terminal maltose binding protein tag. All mutations indicated are substitutions in T4 Rnl2tr + MBP. Data are shown as the mean +/- SEM of at least three independent experiments. * denotes difference in means p < 0.01

The indicated mutations were introduced into T4 Rnl2tr singly or in combination. Mutant ligases were produced as N-terminal Maltose Binding Protein (MBP) fusion proteins in *E. coli *and purified by amylose and Q Sepharose chromatography (Figure 3A).

Variant truncated ligases were examined for their ability to promote the intermolecular ligation of 5'-adenylated 17-mer DNA adapters to 5'-FAM-labeled 31-mer RNA oligonucleotides. This experiment measures ligation step 3 - phosphodiester bond formation. All mutants had strand-joining activity, except the R55K K225R ligase (Figure 3B).

T4 Rnl2tr, T4 Rnl2tr fused to the MBP tag (T4 Rnl2tr+MBP), and R55K displayed the same extent of strand-joining after one hour of incubation, at which ~10% of FAM-RNA was ligated. The extent of strand-joining (~5%) promoted by the K227Q and K225R mutants of the fusion construct was significantly less that of the wild-type T4 Rnl2tr.

The double mutants R55K K227Q and K225R K227Q gave the same or a greater yield of the strand joining reaction product (11% and 5%, respectively) than did their respective single mutants, and the activity of the R55K K227Q mutant was similar to T4 Rnl2tr (Figure 3B). In contrast, the R55K K225R mutant failed to perform the reaction (< 0.1% of substrate ligated). Surprisingly, the triple mutant retained the ability to ligate its substrates and had a similar efficiency to the K225R K227Q ligase (5% of substrate ligated).

### Effect of pH on strand-joining activity

We next examined whether the conservative active site mutations that we had introduced changed the pH optimum for intermolecular strand joining activity (ligation step 3). We performed ligation reactions in buffers from pH 5.0 to pH 9.5 containing 10 mM Mg^2+^, 1 mM DTT and either 10 mM Tris-HCl or 10 mM Tris-acetate. The substrates for the assays were 5'- FAM-labeled 31-mer RNA and 5'-adenylated 17-mer DNA.

**Figure 4:**
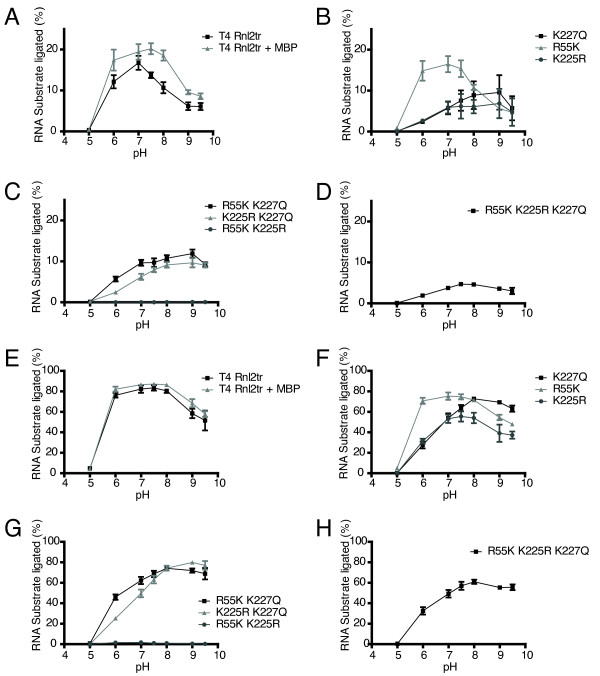
**Effect of pH on ligase intermolecular strand-joining activity**. **(A-D) **Intermolecular strand-joining reactions were carried out with 10 pmol 5'-adenylated 17mer DNA, 5 pmol 31-mer 5'-FAM-labeled RNA acceptor, and ligase (1 pmol) for 1 hour at 25°C to assess the effect of pH on ligation efficiency. Ligation efficiency was determined by resolving the material in the reactions on denaturing 15% acrylamide gels and quantifying the amount of ligation product versus input nucleic acid. **(E-H) **Intermolecular strand-joining reactions were carried out with 10 pmol 5'-adenylated 17-mer DNA, 5 pmol 31-mer 5'-FAM-labeled RNA acceptor, and ligase (13.8 pmol) for 1 hour at 25°C to assess the effect of pH on ligation efficiency. Rnl2tr = T4 RNA ligase 2 truncated, Rnl2tr + MBP = T4 RNA ligase 2 truncated attached to an N-terminal maltose binding protein tag. All mutations indicated are substitutions in T4 Rnl2tr + MBP. Data are shown as the mean +/- SEM of at least three independent experiments.

Under multiple turnover conditions, all proteins were active (except the R55K K225R mutant), but their pH optima differed (Figure 4A-D). We observed that the optimal pH for T4 Rnl2tr is 7.0, where 1 pmol of enzyme ligated 17% of the FAM-labeled RNA, (equivalent to 0.8 pmol of the final product in one hour). All mutations, with the exception of R55K exhibited reduced yield of ligation products under all pH conditions tested as compared to T4 Rnl2tr at its optimum. Variants containing K227Q, or K225R either singly (Figure 4B) or in combination with other substitutions (Figure 4C) had optima shifted toward higher pH. As shown in Figure 4D, the triple mutant had an optimum efficiency between pH 7.0 and 7.5 and the lowest strand-joining activity (5% of input ligated after 1 hour).

Single turnover assays (in enzyme excess) were performed to mimic common usage conditions for T4 Rnl2tr (Figure 4E-H). The assays were identical to the multiple turnover reactions, except for the amount of ligase was increased to 13.8 pmol. T4 Rnl2tr was active over a large pH range from 6.0 to 8.0. This was in contrast to the narrower range observed under multiple turnover conditions (compare Figure 4A to 4E). T4 Rnl2tr+MBP had the same profile (Figure 4E). As shown in Figure 4F, the single mutants had differing pH ranges in which they were maximally active: R55K was most efficient between pH 6.0 to 8.0, while K225R was most efficient between pH 7.0-8.0. The K227Q mutant was most active between pH 8.0 and 9.0. As we observed under single turnover conditions, the double and triple mutants had higher strand-joining activity in higher pH conditions as compared to T4 Rnl2tr (Figure 4G) with the least accumulation of ligation product observed with the triple mutant (Figure 4H). From a practical standpoint, these data are instructive in that all of the ligases perform the strand-joining reaction efficiently when in high concentration in buffer with pH 7.5-8.0.

### Effect of PEG 8000 on single turnover strand-joining reactions

**Figure 5:**
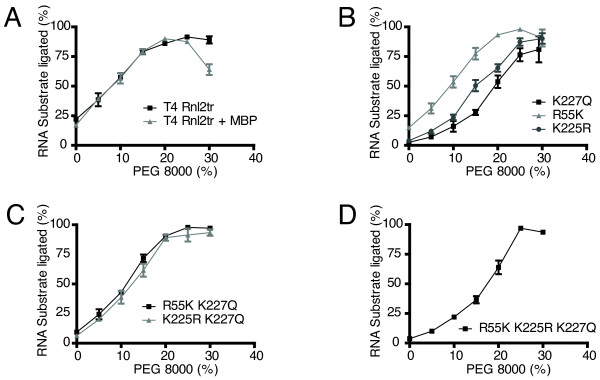
**Effect of PEG 8000 on ligase intermolecular strand-joining activity**. Strand-joining reactions were carried out with 10 pmol 5'-adenylated 17-mer DNA, 5 pmol 31-mer 5'-FAM-labeled RNA acceptor, ligase (13.8 pmol), and varying amounts of PEG 8000 for 1 hour at 25°C to assess the effect of PEG on ligation efficiency. Ligation efficiency was determined by resolving the material in the reactions on denaturing 15% acrylamide gels and quantifying the amount of ligation product versus input nucleic acid. Rnl2tr = T4 RNA ligase 2 truncated, Rnl2tr + MBP = T4 RNA ligase 2 truncated attached to an N-terminal maltose binding protein tag. All mutations indicated are substitutions in T4 Rnl2tr + MBP. Data are shown as the mean +/- SEM of at least three independent experiments.

Polyethylene glycol (PEG) is known to stimulate ligation reactions for the T4 RNA ligases [10]. We examined the activity of the mutants at different PEG 8000 concentrations for intermolecular ligation of 5'-adenylated DNA to 3'-OH RNA (Figure 5).

In agreement with our previous observations, strand joining of 5'-FAM-labeled 31-mer RNA to 5'-adenylated 17-mer DNA adapter was stimulated as PEG 8000 concentration increased up to 25% for T4 Rnl2tr [11]. T4 Rnl2tr+MBP showed an identical response up to 25% PEG (Figure 5A). We did not observe further stimulation when PEG concentration was increased beyond 25%, and handling concentrations of PEG greater than 25% was difficult because of the high viscosity of the reactions.

Overall, the single and multiple mutants displayed stimulated strand joining activity with increased concentration of PEG 8000 (Figure 5B-D). Under maximal PEG stimulation, all variants tested were able to convert nearly 100% of the substrate to ligated form.

### Strand-joining activity over time

**Figure 6:**
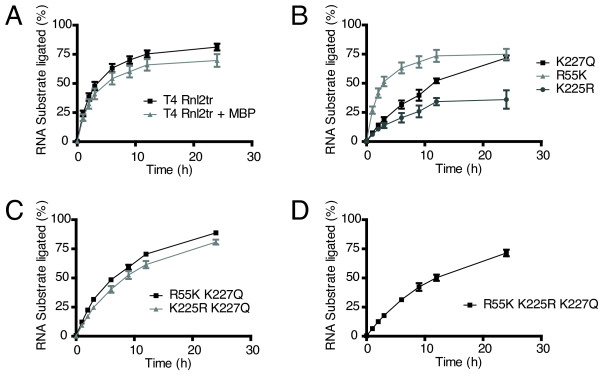
**Analysis of intermolecular strand-joining over time**. Strand-joining reactions were carried out with 10 pmol 5'-adenylated adapter, 5 pmol 31-mer 5'-FAM-labeled RNA acceptor, and ligase (1 pmol) over a span of 24 hours at 25°C to assess the progress of ligation reactions. Ligation efficiency was determined by resolving the material in the reactions on denaturing 15% acrylamide gels and quantifying the amount of ligation product versus input nucleic acid. Rnl2tr = T4 RNA ligase 2 truncated, Rnl2tr + MBP = T4 RNA ligase 2 truncated attached to an N-terminal maltose binding protein tag. All mutations indicated are substitutions in T4 Rnl2tr + MBP. Data are shown as the mean +/- SEM of at least three independent experiments.

We performed time course experiments to monitor the progression of intermolecular strand-joining reactions under multiple turnover conditions using a 5'-FAM-labeled 31-mer RNA and a 17-mer 5'-adenylated DNA adapter (Figure 6). The amount of ligated product was calculated (in % of input) at different time points between 0 and 24 hours. All ligase variants tested, except K225R, ligated ~75% of the input RNA after 24 h. The K225R variant accumulated ~36% of ligated product. Given the input concentration of ligase, these experiments indicate an average of 4 ligation events per enzyme molecule over the course of the reaction for all variants, except K225R which catalyzed 2 events.

Ligation reactions containing K225R accumulated significantly less ligated products than T4 Rnl2tr (Figure 6A and 6B, and Table 1). After one hour of ligation, ligated products accumulated to 6.1 +/-1% of maximum when incubated with K225R as compared to 24 +/- 2.3% of maximum when incubated with T4 Rnl2tr (mean +/- SEM, p < 0.01). After 2, 3, 6, 9, 12 and 24 hours, ligated products accumulated to 11 +/- 1%, 14 +/- 2%, 21 +/- 4%, 26 +/- 5%, 34 +/- 3%, and 36 +/- 8% when incubated with K225R as compared to. 39 +/-3%, 48 +/- 3%, 64 +/- 3%, 70 +/- 3%, 76 +/- 3%, 52 +/- 2%, and 76 +/- 3% when incubated with T4 Rnl2tr (mean +/- SEM, p < 0.001).

**Table 1:** Differences in mean RNA substrate ligated by T4 Rnl2tr mutants over time.

Time (h)	Rnl2tr + MBP	K227Q	R55K	K225R	R55K K227Q	K225R K227Q	R55K K225R K227Q
1	NS	*	NS	**	NS	NS	NS

2	NS	***	NS	***	NS	NS	**

3	NS	***	NS	***	NS	**	***

6	NS	***	NS	***	NS	**	***

9	NS	***	NS	***	NS	NS	***

12	NS	**	NS	***	NS	NS	**

24	NS	NS	NS	***	NS	NS	NS

Mean levels of RNA substrate ligated by T4 Rnl2tr mutants that are significantly different are lower than the corresponding WT T4 Rnl2tr values (see Fig 6). NS = no significant difference detected, * = means differ p < 0.05, ** = means differ p < 0.01, *** = means differ p < 0.001 as determined by two-way ANOVA followed by Bonferroni post-tests for comparison to T4 Rnl2tr.

We noted that K227Q accumulated ligation products significantly more slowly than Rnl2tr, or Rnl2tr+MBP, but accumulated to the same degree after 24 hours of incubation. After one hour, ligated products accumulated to 7.5 +/-0.6% of maximum when incubated with K227Q vs. 24 +/- 2.3% of maximum when incubated with T4 Rnl2tr (mean +/- SEM, p < 0.05). After 2, 3, 6, and 9 hours, ligated products accumulated to 14 +/- 1%, 19 +/- 2%, 32 +/- 4%, and 40 +/- 5% when incubated with K227Q vs. 39 +/-3%, 48 +/- 3%, 64 +/- 3%, 70 +/- 3%, and 76 +/- 3% when incubated with T4 Rnl2tr (mean +/- SEM, p < 0.001). After 12 hours, ligated products accumulated to 52 +/- 2% of maximum when incubated with K227Q, while products accumulated to 76 +/- 3% of maximum for T4 Rnl2tr (mean +/- SEM, p < 0.01). Accumulated ligation products were not significantly different after 24 of incubation 72 +/- 2% vs. 81 +/- 3%, for K227Q vs. T4 Rnl2tr (mean +/- SEM, p > 0.05) (Figure 6A and 6B, and Table 1).

Interestingly, combining R55K and K227Q increased the accumulation of ligated RNA at earlier time points, and we could detect no difference at any time point comparing R55K K227Q to Rnl2tr.

Combining K225R with K227Q increased ligation product accumulation at earlier time points and the total product accumulated after 24 h as compared to K225R alone (Figure 6C, Table 1). The accumulated product for this mutant was significantly lower after 3 and 6 hours of ligation, but not before of after these time points. After 3, and 6 hours of ligation with the K225R K227Q mutant, ligation products had accumulated to 25 +/- 2%, and 40 +/- 3% if maximum, compared to 48 +/- 3%, and 64 +/- 3% of maximum with T4 Rnl2tr (mean +/- SEM, p < 0.01).

The triple mutant displayed a similar profile to the K227Q mutant for the accumulation of ligation products (Figure 6D andTable 1).

Interestingly, T4 Rnl2tr, T4 Rnl2tr+MBP, the R55K and the K225R mutants did not accumulate additional ligation products after twelve hours of reaction. In contrast, the K227Q, the R55K K227Q and the K225R K227Q, and triple mutants continue to accumulate ligated products over the entire course of the experiment. By the end of the experiment, only the K225R mutant had accumulated significantly less ligated substrate than wild-type T4 Rnl2tr (Figure 6 and Table 1)

Considered together, the results so far established that the introduction of conservative mutations, singly and in combination at positions 55, 225, and 227, in the context of the truncated T4 RNA ligase 2 yielded ligases that could reasonably be used as tools for molecular biology. We next sought to determine whether these ligases had increased performance with respect to the formation of unwanted ligation products.

### Concatemer formation by T4 Rnl2tr variants

Ligation side products such as concatemers and circles are problematic for ligase applications such as high-throughput sequencing library construction. We tested the ability of each ligase to produce desired and undesired products in intermolecular ligation reactions.

Ligation reactions were performed using 5'-PO_4 _22-mer RNA acceptor and 5'-adenylated 17-mer DNA donor. The DNA donor was blocked at the 3'-end by the addition of an -NH_2 _group. Thus the 5'-PO_4 _end of RNA could serve as a ligation donor substrate for joining to the 3'-OH of another RNA 22-mer. Reactions were performed in buffer containing 10 mM Tris HCl pH 7.5, 10 mM Mg^2+^, 1 mM DTT and 12.5% PEG 8000, to maximize ligation efficiency. Expected products were 39 nt in length, and ligation side products were predicted to be 39+22n or 22+22n (where n is a natural number ∈ [1: ∞]) nt in length.

**Figure 7:**
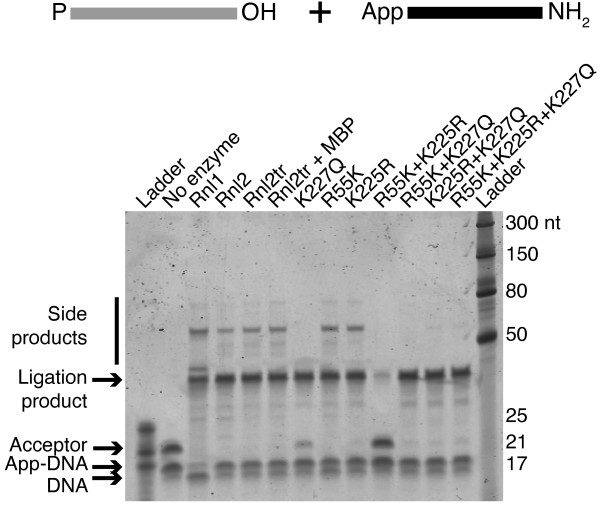
**Assaying the formation of side products by T4 RNA ligases**. Intermolecular strand-joining reactions containing 5'-adenylated adapters, 21-mer 5'-PO_4 _RNA acceptors, and ligase (1 pmol) were incubated at 16°C overnight in the presence of 12.5% PEG 8000. Oligonucleotide substrates are depicted schematically above the gel. Grey lines represent RNA and black lines represent DNA. Products of the reaction were resolved on denaturing 15% acrylamide gels and stained with SYBR Gold. The bands corresponding to the input nucleic acids, the DNA adapter/RNA acceptor ligation product (39 bases), and larger side products are indicated. Ladder = size standard ladder, Rnl1 = T4 RNA ligase 1, Rnl2 = T4 RNA ligase 2, Rnl2tr = T4 RNA ligase 2 truncated, Rnl2 +MBP = T4 RNA ligase 2 truncated attached to an N-terminal maltose binding protein tag. All mutations indicated are substitutions in T4 Rnl2tr + MBP.

The two WT ligases (Rnl1 and Rnl2 in Figure 7), and the wild-type truncated Rnl2 (Rnl2tr and Rnl2tr+MBP) formed the final ligation product (39-mer band), but also formed higher molecular weight species of ~ 60 and 80 nt. For these ligases, the 5'-PO_4 _RNA band was completely absent at the end of the reaction indicating that all the substrate was consumed.

The single K227Q mutation produced only the desired ligation product, whereas ligation side products were observed for R55K and K225R. The R55K+K225R mutant showed low levels of accumulated ligation product of the correct size, consistent with the low activity we observed in earlier experiments. Other multiple mutants, all of which contained the K227Q mutation, formed dramatically reduced levels of undesired ligation products. Together, these observations correlate K227 with the formation of undesired ligation products.

### Adapter deadenylation

Our ligation conditions did not contain ATP, yet we continually observed the accumulation of ligation side products that could only be explained by the concatemerization of RNA inputs that would require the adenylation of 5'-PO_4 _ends. In the absence of exogenous ATP, one possible source of adenylyl groups in our experimental system is ligation reaction 2 - transfer of AMP from the ligase active site to the 5'-PO_4 _of the donor oligonucleotide - running in reverse. That is, the transfer of adenylyl groups from the adenylated donor substrate to the catalytic lysine in the active site of the ligase.

**Figure 8:**
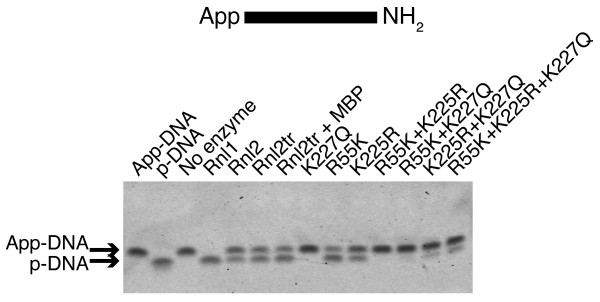
**Deadenylation activity of T4 RNA ligase 2 truncated mutants**. 5'-adenylated DNA adapters were incubated with an excess of ligase (13.8 pmol), and 12.5% PEG 8000 at 16°C overnight. Oligonucleotide substrates are depicted schematically above the gel. The contents of each sample were resolved on denaturing 15% acrylamide gels and stained with SYBR Gold to visualize nucleic acid. Deadenylation of the DNA adapter (loss of 5'-App) is indicated by a band shift of ~1 nt towards the bottom of the gel. Rnl1 = T4 RNA ligase 1, Rnl2 = T4 RNA ligase 2, Rnl2tr = T4 RNA ligase 2 truncated, Rnl2 +MBP = T4 RNA ligase 2 truncated attached to an N-terminal maltose binding protein tag. All mutations indicated are substitutions in T4 Rnl2tr + MBP.

We tested the ability of T4 Rnl2tr and variants to remove the AMP from 5'-adenylated DNA oligonucleotides by incubating these substrates overnight in the absence of acceptor under single turnover ligation reaction conditions containing 12.5% PEG 8000 (Figure 8). After incubation, reactions lacking enzyme had only one band corresponding to the 5'-adenylated DNA oligonucleotide indicating that it was stable during the assay. In contrast, when incubated with T4 Rnl1, we observed a single band with lower molecular weight that co-migrated with 5'-PO_4 _DNA adapter. We interpreted this result to indicate that the AMP was completely removed from the 5'-adenylated DNA adapter.

Adenylated substrates incubated with T4 Rnl2 WT, Rnl2tr and T4 Rnl2tr+MBP migrated as two bands; one corresponding to the input, and the other that migrated at the same position as the 5'-PO_4 _adapter. We interpret this to indicate that these enzymes were able to remove the adenylyl groups from some of the substrate. R55K and K225R mutants similarly converted the adenylated substrates into 2 species. On the other hand, the adenylated DNA adapters incubated with K227Q migrated as the higher molecular weight intact species. Adenylated DNA adapters incubated overnight with the multiply mutated T4 Rnl2tr variants were largely unchanged. These results correlate the deadenylation activity of T4 Rnl2 with K227.

### AMP transfer by variant ligases

The observation that incubation of T4 RNA ligases with adenylated oligonucleotides could result in changes in their migration consistent with deadenylation led us to directly monitor the fate of the AMP group in question. To do so we followed AMP transfer during the ligation reaction using ^32^P-α-AMP-labeled adenylated DNA substrates and 5'-PO_4 _RNA.

### Concatemers

**Figure 9:**
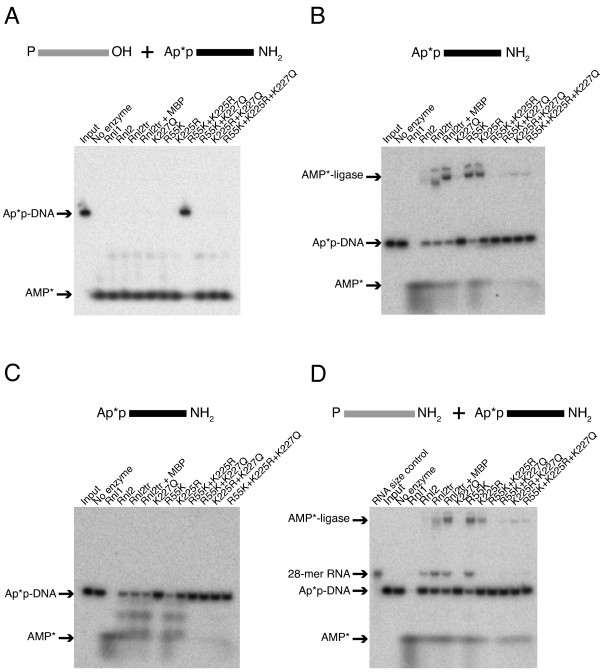
**Following AMP during ligation reactions with T4 RNA ligases**. **(A) **22-mer DNA adapters were 5'-adenylated with α-^32^P-labeled ATP (see materials and methods). Intermolecular strand-joining reactions containing 10 pmol radiolabeled DNA adapter, 5 pmol 21-mer 5'-PO_4 _RNA acceptor, and ligase (1 pmol) were incubated overnight at 16°C in the presence of PEG 8000. Reaction products were resolved on a denaturing 15% acrylamide gel and radioactive molecules were visualized by exposure to Phosphor screens. The resulting products were either free AMP in solution (AMP*) or the adapter remaining adenylated (Ap*p-DNA). Oligonucleotide substrates are depicted schematically above the gel. Grey lines represent RNA and black lines represent DNA. P* denotes ^32^P-phosphate. **(B) **Determining the fate of AMP upon T4 RNA ligase-dependent deadenylation. Reactions containing radiolabeled DNA adapter (10 pmol) and ligase (14 pmol) were incubated overnight at 16°C in the presence of 12.5% PEG 8000. Oligonucleotide substrates are depicted schematically above the gel. P* denotes ^32^P-phosphate. Reaction products were resolved and visualized as in (A). The resulting products were either free AMP in solution (AMP*), the adapter remaining adenylated (Ap*p-DNA), or AMP covalently bound to the ligase (AMP*-ligase). The lane labeled input contains only Ap*p-DNA. **(C) **Reactions identical to those in (B) were treated with Proteinase K prior to gel electrophoresis and detection. **(D) **Reactions containing 10 pmol radiolabeled DNA adapter, 5 pmol 28-mer [5'-PO_4_, 3'-blocked] RNA acceptor, and ligase (1 pmol) were incubated, resolved and detected as in (A). The resulting products were either free AMP in solution (AMP*), adenylated adapter (Ap*p-DNA), or Ap*p-28-mer RNA. The lane labeled RNA size control contains 5'-^32^PO_4 _RNA, and the lane labeled input contains only Ap*p-DNA. Oligonucleotide substrates are depicted schematically above the gel. Grey lines represent RNA and black lines represent DNA. P* denotes ^32^P-phosphate. In all panels, Rnl1 = T4 RNA ligase 1, Rnl2 = T4 RNA ligase 2, Rnl2tr = T4 RNA ligase 2 truncated, Rnl2 +MBP = T4 RNA ligase 2 truncated attached to an N-terminal maltose binding protein tag. All mutations indicated are substitutions in T4 Rnl2tr + MBP.

Intermolecular ligation reactions were carried out using 10 pmol of 5'-α-^32^P-adenylated DNA adapter and 5 pmol of 5'-PO_4 _RNA overnight with one pmole of ligase (Figure 9A). When resolved by urea PAGE, the negative control reaction containing no enzyme displayed a single band that migrated at 22 nucleotides indicating the stability of the α- ^32^P-AMP attached to the DNA adapter (Ap*p-DNA) over the course of the experiment. The majority of the radioactivity detected was concentrated at the bottom of the gel for all of the reactions containing ligase except for the R55K K225R reaction where the majority of signal was observed to co-migrate with a 22-mer length. This was consistent with the inactivity of this variant observed in other experiments. We interpret the results from the other ligase variants to indicate that, by the end of the reaction, the radioactive AMP had been released as a nucleotide into the reaction mixture.

These results are consistent either with ligation of the adenylated DNA to the intended 3'-OH end of the RNA, or with AMP addition to the unintended 5'-PO_4 _RNA substrate that results in concatemers, followed by phosphodiester bond formation. In both scenarios, α-^32^P-AMP would be released into the reaction mixture.

### Deadenylation

We performed deadenylation activity assays of the mutants by performing ligation reactions in the absence of the RNA acceptor (Figure 9B). In control reactions containing no-enzyme, only the input 22-mer oligonucleotide (Ap*p-DNA) was evident, indicative of the stability of the 5'-adenylated DNA adapter.

Radioactive signal that migrated with the adenylated adapter was absent from the reaction incubated with T4 Rnl1. Instead, the entirety of the signal co-migrated with free AMP. We interpret this to mean that T4 Rnl1 has the ability to both remove the α-^32^P AMP from the adenylated adapter and to also release it into the reaction mixture. When reactions containing T4 Rnl2, Rnl2tr and Rnl2tr+MBP were resolved, they had reduced signal that co-migrated with the intact adenylated adapter. Full-length T4 Rnl2 had an increased radioactive signal that co-migrated with AMP. We interpret this result to indicate that full-length T4 Rnl2, like T4 Rnl1 has the ability to remove the AMP from the adenylated adapter and to release it into solution. In contrast to T4 Rnl1, in reactions that contained T4 Rnl2, Rnl2tr, and Rnl2tr+MBP, we observed radioactive signal that migrated with higher molecular weight than the intact adenylated adapter, as well as a smaller amount of signal that co-migrated with AMP. The high molecular weight signal that we observed was consistent with the covalent attachment of α-^32^P AMP to the ligase itself (AMP*-ligase).

Adenylated substrate incubated with T4 Rnl2tr K227Q remained largely unchanged, and we did not observe radioactive signal that co-migrated with AMP or with high molecular weight. Reactions containing R55K and K225R yielded signals co-migrating with the adenylated DNA adapter and with higher molecular weight species. As with the unmutated ligases, we interpret this result to indicate that these variants are able to remove the adenylyl group from the adenylated adapters and that it remains attached to the ligase. As found for K227Q, reactions containing the ligases with multiple mutations, showed the majority of ^32^P signal co-migrating with the adenylated adapter. Treatment of the ligation reactions with Proteinase K shifted the high molecular weight radioactive signal so that it migrated with lower molecular weight, suggesting that the higher MW band is indeed ligase-^32^P-AMP since ligases are sensitive to Proteinase K digestion. (Figure 9C).

### AMP transfer from ligation donor to RNA 5'-PO_4_

To establish that AMP could be transferred from an adenylated DNA adapter to an RNA 5'-end, we incubated radioactively adenylated DNA adapters (Ap*p-DNA) with 5'-PO_4 _RNA that was blocked at its 3'-end (Figure 9D). Blocking the 3'-end of the RNA prevented strand-joining. In the absence of enzyme, we observed radioactive signal corresponding to the adenylated adapter. In reactions that contained Rnl1, the entirety of the radioactive signal migrated with low molecular weight corresponding with AMP. In reactions incubated with Rnl2, Rnl2tr, Rnl2tr+MBP, and R55K, we observed radioactive signal that co-migrated with 28-mer 3'-blocked RNA. We additionally observed higher molecular weight AMP*-ligase adducts for Rnl2tr, Rnl2tr+MBP, R55K and K225R. We did not observe radioactive signal corresponding to the 28-mer RNA for the inactive ligase R55K+K225R, or for the active ligases containing K227Q.

Taken together, we interpret these results to directly demonstrate the reversal of ligation reaction step 2 - transfer of adenylyl groups from adenylated oligonucleotide donors to the ligase, and from the ligase to RNA 5'-PO_4 _ends, or to generate free AMP. Furthermore our observations are consistent with the requirement of a lysine residue at position 227 for this reverse reaction to occur.

## Discussion

In this work, we introduced conservative active-site mutations in T4 Rnl2tr to determine their characteristics, especially as they relate to formation of unwanted ligation products. We could detect no apparent difference in the strand-joining activity of T4 Rnl2tr and the T4 Rnl2tr+MBP, and interpret this to mean that the MBP tag does not influence strand-joining activity.

T4 Rnl2 has six characteristic motifs in the N-terminus which are involved in catalysis: motif I, Ia, III, IIIa, IV and V [6]. During ligation step 1, the AMP is covalently bound to the K35 in motif I. The adenine of the adenylyl group is close to F119 (motif IIIa), while the ribose interacts with E99 (motif III) and R55 (motif Ia) via hydrogen bonds with the 3' and 2' carbons. The phosphate group is also hydrogen-bonded with K227 (motif V). Between step 1 and 2, the active site is remodeled: the AMP phosphate is now coordinated with K35 (motif I) and K225 (motif V). A conformational change takes place so that R55 no longer coordinates the ribose O3' but gains contacts with the two oxygens of the 5'-PO_4 _RNA. R55 is involved in the orientation and the recognition of the 5'-PO_4 _end [4].

Based on mutational studies of full length T4 Rnl2 [6], and structural studies of the adenylyl transferase domain [5], R55, K225, and K227 were implicated as key catalytic residues for the enzyme adenylation step. We have extended these findings by demonstrating that of the three, only K227 is necessary for the reverse reaction of ligation step 2 (deadenylation of AppDNA). Thus K227 is a catalytic residue for adenylyltransferase both in the forward and backward direction.

Co-crystal studies revealed two structural forms containing T4 Rnl2 with 5'-adenylated ligation donor and a 3'- end ligation acceptor [4]. Significant remodeling of the active site between step 2 product formation and the step 3 substrate binding was observed. R55 no longer interacts with the ligation donor 5'-PO_4 _oxygen, and K227 and K35 cease to interact with the AMP phosphate. The phosphoanhydride region of the ligation donor changes its orientation to interact with the 3'-OH RNA with more optimal geometry for strand joining [4]. Since K227 also plays a role in coordinating the AMP oxygen and in step 2-3 structural rearrangements, we infer that substitution of K227Q explains both the lack of reverse step 2 and the slow phosphodiester bond formation (step 3). The slower rate observed for step 3 may occur because substitution of the side chain of lysine at position 227 with glutamine interferes with re-orienting the AppDNA relative to the RNA 3'-OH to a configuration conducive to phosphodiester bond formation.

All of the T4 Rnl2tr mutants were active in strand-joining reactions except the R55K K225R ligase. This variant was also inactive for reversal of ligation step 2. We did not detect any evidence of protein instability for this mutant. However, neither the R55K, or K225R single mutants nor the R55K K225R K227Q triple mutant showed a loss of activity similar to the R55K K225R double mutant. This R55K K225R double mutation may either severely disrupt the function of the active site by modifying the interaction between the substrates, or cause protein misfolding.

Previous studies demonstrated equivalent strand-joining activity when R55K was compared to full-length wt T4 Rnl2 [6]. We report similar observations in the context of T4 Rnl2tr. In our studies K227Q and K225R ligases displayed reduced strand-joining activity, also consistent with that study. Curiously, the R55K K227Q had similar strand joining activity to T4 Rnl2tr, suggesting that the R55K mutation is able to compensate for the reduced activity of the K227Q mutant.

Studying substrate turnover for the ligase variants illustrated that on a molar basis, except for the K225R mutant, all ligase variants could perform the strand-joining reaction to the same extent as T4 Rnl2tr. This suggests to us that the mutations do not decrease the final efficiency of the ligases; rather the speed of the strand-joining reaction is altered for these mutants. The K225R mutation seems to be detrimental to the enzyme since its turnover was reduced in comparison to the other mutants. However, the addition of PEG 8000 demonstrated that it possible to restore K225R activity, since we observed strand joining of more than 80% of FAM-labeled substrate with high concentrations of the crowding agent. PEG 8000 appeared to increase the rate of the strand joining reaction for all variants tested. The reduced total turnover observed for the K225R variant may therefore have resulted from the combined effects of slow turnover and enzyme stability in our time course experiments.

We studied the ability of the variant ligases to form concatemers and circles from 5'-PO_4 _RNA and the ability to deadenylate a 5'-adenylated DNA adapter. Our results demonstrate that only the K227Q and the R55K K227Q could prevent the formation of concatemers while retaining the ability to perform the strand-joining reaction. Other ligases formed ligation side products by the end of 24-hour experiments. The K225R K227Q and R55K K225R K227Q ligases produced fewer side products than the T4 Rnl2tr. The K227Q and the R55K K227Q mutants are the only active variants that completely prevented adapter deadenylation, whereas the K225R K227Q and the triple mutants have deadenylation activity, albeit reduced. Thus, the ligases that have impaired or no ability to remove the AMP from the 5'-adenylated DNA adapter also produce fewer ligation side products.

By following α-^32^P-AMP in the ligation reaction we sought to determine whether the reverse reaction of ligation step 2 could occur. Our results show that AMP was released into the reaction mixture for all of the active ligases. However in the absence of a ligation acceptor, radioactive signal from adenylated DNA was markedly reduced (donor substrate destroyed). This was evident for all ligases, except for active variants that had K227Q. For some ligases, a higher molecular weight signal was observed. Since the reaction mixture contained only radioactive adapter, buffer and ligase, we attribute the high molecular weight signal to ^32^P AMP-protein intermediate. Digestion of the reactions with proteinase K confirmed this assertion. We conclude that the reverse reaction occurred in the mixture for all of the active T4 Rnl2tr variants except for the K227Q and the R55K K227Q mutants. By introducing 5'-PO_4_, 3'-blocked RNA into the ligation reactions, we directly followed the radioactive adenylate transfer from adenylated DNA adapter to 5'-PO_4 _RNA. Thus concatemers and circles may be formed by T4 Rnl2tr because of its ability to transfer the AMP from the 5'-adenylated DNA adapter to itself, and then to a new 5'-PO_4 _RNA. Moreover, the deletion of the C-terminal domain from the full length T4 Rnl2 is not sufficient to prevent the transfer of the AMP: only the K227Q mutation completely prevented this reaction in the context of the truncated ligase.

## Conclusions

T4 Rnl2 is now commonly used in RNA research, notably for the identification of small RNAs by generating libraries for sequencing with high-throughput sequencing technology. The mutations that we tested in the context of the truncated enzyme have established the utility of variants including K227Q [8], in reducing unwanted side products, and shed light on the roles of important active site residues and their formation.

T4 Rnl2tr K227Q produces different results in small RNA profiling experiments by microarrays as compared to T4 Rnl1 or T4 Rnl2tr [12]. It seems likely that the reduction of ligation side products should improve RNA sequencing library quality, perhaps resulting in the generation of data that more accurately reflects the composition of starting RNA pools.

## Methods

### Oligonucleotides

DNA and RNA oligonucleotides were produced and purified by Integrated DNA Technologies (Coralville, IA). 5'-adenylated Universal MicroRNA cloning linker (referred to in the text as 17-mer adenylated DNA) was produced by New England Biolabs (Ipswich, MA). Sequences of oligonucleotides are listed in Table 2.

**Table 2:** Oligonucleotides used in this study

Name	Sequence	Properties
Universal miRNA Cloning Linker (17-mer DNA adapter)	CTGTAGGCACCATCAAT	5'-rApp 3'-NH_2_

5'-FAM labeled 31-mer RNA	rArGrUrCrGrUrArGrCrCrUrUrUrArUrCrCrGrArGrArUrUrCrArGrCrArArUrA	5'-FAM

22-mer DNA adapter	TCGTATGCCGTCTTCTGCTTGT	5'-PO_4_ 3'-NH_2_

5'PO_4 _21-mer	rArGrCrArGrUrGrGrCrUrGrGrUrUrGrArGrArUrUrU	5'-PO_4_

28-mer RNA	rArCrArArGrCrArGrArArGrArCrGrGrCrArUrArCrGrArUrArUrUrGrC	5'-PO_4_ 3'-NH_2_

### Adenylation of DNA oligos

225 pmol of a 22-mer DNA oligo with 5'-PO_4 _and 3'-Amino modifications (see Table 2) was adenylated by 225 pmol of mutant Mth ligase (kindly provided by A. Zhelkovsky, New England Biolabs.) in a 30 μL reaction (50 mM Bis-Tris propane pH 8.0, 5 mM MgCl_2_, 0.5 mM ATP). The buffer was supplemented with 15 fmol [α-^32^P]ATP (Perkin Elmer, Waltham, MA) and the reaction was carried out for 2 hours at 65°C. Reactions were heated to 95°C for 5 min and then treated with 20 μg of Proteinase K (New England Biolabs) for 30 min at 37°C to inactivate the ligase. The samples were then passed through a G-25 column (GE Life Sciences, Piscataway, NJ) to remove unincorporated ATP, and loaded onto a denaturing 20% acrylamide gel for purification. The band corresponding to adenylated oligo was excised and eluted overnight at room temperature in 10 mM Tris-HCl pH 8.0. A 0.45 μm microcon column (Millipore, Billerica, MA) was used to remove pieces of gel and the adenylated adapter was precipitated in ethanol using 5 μg of linear acrylamide (AMRESCO, Solon, OH) to increase the visibility of the pellet. After precipitation, the pellet was suspended in nuclease-free water (Ambion, Austin, TX).

### Expression and purification of mutant T4 RNA ligase 2 truncated (Rnl2tr) proteins

Mutations were introduced into the T4 Rnl2tr coding sequence by site-directed mutagenesis using the Phusion Site-directed Mutagenesis Kit (New England Biolabs). The coding sequences were inserted into a pMAL-C4X vector (New England Biolabs) in order to incorporate a maltose binding protein (MBP) tag at the N-terminus. Resulting plasmids were transformed into *E. coli *New England Biolabs Turbo cells for cloning and sequencing and then moved into a T7 expression strain for protein production.

For protein production and purification, 2 L of LB medium with 100 μg/mL ampicillin was inoculated with 3 mL of cells from an overnight culture and grown at 37°C. When an OD_600 _of 0.5 was reached 0.5 M, IPTG was added to a final concentration 0.3 mM to induce expression of the MBP-fusion protein, and cells were incubated for an additional 16 hours at 16°C. Cells were harvested by centrifugation, resuspended in column buffer (200 mM NaCl, 20 mM Tris-HCl pH 7.5, 1 mM EDTA), and frozen at -20°C overnight. The cells were thawed in an ice water bath, diluted 2-fold in column buffer and sonicated with 3 repetitions of 1 minute continuous pulsing on a Heat Systems-Ultrasonics Inc. cell disruptor. The resulting lysate was loaded on a 90 mL column packed with High-Flow Amylose Resin (New England Biolabs) with a flow rate of 2.5 mL/min. The column was washed with 10 column volumes of wash buffer (500 mM or 1 M NaCl, 20 mM Tris-HCl pH 7.5, 1 mM EDTA), and eluted in 40 5 mL fractions with elution buffer (200 mM NaCl, 20 mM Tris-HCl pH 7.5, 1 mM EDTA, 10 mM maltose). Samples of fractions were analyzed by SDS-PAGE and fractions containing protein were pooled for a second round of purification on a Q Sepharose column (GE Healthcare). The column and wash buffer for Q Sepharose purification consisted of 50 mM NaCl, 20 mM Tris-HCl pH 7.5, 1 mM EDTA. Elution was carried out by increasing the NaCl concentration by a gradient from 50 mM to 1 M over the course of 10 column volumes. Fractions containing protein of interest were pooled and protein concentration was determined by a Bio-Rad Dc protein assay (Bio-Rad, Hercules, CA) after overnight dialysis (100 mM NaCl, 10 mM Tris-HCl pH 7.5, 0.1 mM DTT, 0.1 mM EDTA, 50% glycerol). Successful purification was monitored by SDS-PAGE followed by Coomassie blue staining (Life Technologies, Carlsbad, CA). T4 RNA ligase 1, T4 RNA ligase 2, T4 RNA ligase 2 Truncated and, T4 RNA ligase 2 Truncated K227Q were obtained from New England Biolabs.

### Ligation reactions

Ligation reactions were carried out in ligation buffer (50 mM Tris-HCl pH 7.5, 10 mM MgCl_2_, 1 mM DTT). For variable pH reactions, Tris-acetate was substituted for Tris-HCl at pH 5.0 and 6.0. Reactions contained 10 pmol of 5'-adenylated DNA adapter and 5 pmol of an RNA acceptor unless indicated otherwise. The reactions used for Figure 7, 8, and 9 also contained 12.5% (w/v) PEG 8000 (Promega, Madison WI). Reactions were performed under multiple turnover conditions where the amount of ligase was limiting (1 pmol) or under single turnover conditions where ligase was provided in excess (13.8 pmol), as indicated. Some reactions were treated with 5 μg Proteinase K (New England Biolabs) for 30 min at 37°C. Products of the reactions were resolved by PAGE on 15% acrylamide gels containing 1xTBE and urea, stained with SYBR Gold (Life Technologies) or exposed to a Phosphor screen (GE Life Sciences), and visualized on a Typhoon 9400 variable mode imager (GE Life Sciences). Nucleic acid bands were quantified by Quantity One software (Bio-Rad) and the percentage of ligated product was determined by dividing the intensity of the ligated product band by the sum total of the ligated product band and input acceptor band. Protein and ssRNA molecular weight markers were from New England Biolabs. Radioactive size standard 28-mer RNA (Table 2) was labeled using T4 PNK (New England Biolabs) with [γ-^32^P]ATP (Perkin Elmer).

### Statistics

Statistical analyses were performed using GraphPad Prism. For strand-joining activity (Figure 3), one-way ANOVA followed by Dunnett's Multiple Comparison test was used compare RNA substrate ligated in reactions containing variant T4 Rnl2 to wild-type T4 Rnl2. For strand-joining activity over time (Figure 6), the data were analyzed using two-way ANOVA followed by Bonferroni post-tests comparing RNA substrate ligated in reactions containing variant T4 Rnl2 to wild-type T4 Rnl2 at each time point.

## Authors' contributions

SV created and purified the T4 RNA ligase 2 truncated variants in this study, performed experiments, analyzed the data and participated in the writing of the manuscript. RF performed experiments and analyzed data. DM made the original observation of concatemer formation by T4 RNA ligases. FZ aided in experimental design and data analysis. GBR conceived of the study, designed the experiments, coordinated and wrote manuscript. All authors have read and approved the final manuscript.
